# A Unitary ESPRIT Scheme of Joint Angle Estimation for MOTS MIMO Radar

**DOI:** 10.3390/s140814411

**Published:** 2014-08-07

**Authors:** Chao Wen, Guangming Shi

**Affiliations:** State Key Laboratory of Integrated Services Networks, School of Electronic Engineering, Xidian University, No.2 Taibai South Road, Xi'an 710071, China; E-Mail: candieswen@gmail.com

**Keywords:** MOTS MIMO, DOD and DOA estimation, OTS, Unitary ESPRIT

## Abstract

The transmit array of multi-overlapped-transmit-subarray configured bistatic multiple-input multiple-output (MOTS MIMO) radar is partitioned into a number of overlapped subarrays, which is different from the traditional bistatic MIMO radar. In this paper, a new unitary ESPRIT scheme for joint estimation of the direction of departure (DOD) and the direction of arrival (DOA) for MOTS MIMO radar is proposed. In our method, each overlapped-transmit-subarray (OTS) with the identical effective aperture is regarded as a transmit element and the characteristics that the phase delays between the two OTSs is utilized. First, the measurements corresponding to all the OTSs are partitioned into two groups which have a rotational invariance relationship with each other. Then, the properties of centro-Hermitian matrices and real-valued rotational invariance factors are exploited to double the measurement samples and reduce computational complexity. Finally, the close-formed solution of automatically paired DOAs and DODs of targets is derived in a new manner. The proposed scheme provides increased estimation accuracy with the combination of inherent advantages of MOTS MIMO radar with unitary ESPRIT. Simulation results are presented to demonstrate the effectiveness and advantage of the proposed scheme.

## 1. Introduction

Multiple-input multiple-output (MIMO) radar is a radar system transmitting multiple linearly independent waveforms which enables joint data processing received by multiple receive antennas. As an emerging field of radar research, MIMO radar has attracted intensive research [1–5]. In terms of the antenna configuration, MIMO radars can be divided into two types: widely separated antennas [3] or colocated antennas [4,5] which includes bistatic and monostatic MIMO radar with transmit and receive antennas closely spaced. Over conventional phased-array counterparts, MIMO radars offer unique advantages, such as extra degrees of freedom offered by waveform diversity [6], higher resolution and better parameter identifiability [7], and a larger virtual or effective aperture than the real aperture [8].

However, the advantages offered by the MIMO radar come at the price of sacrificing transmit directional gain at the transmit array. Since MIMO radar transmits each orthogonal waveform omni-directionally, it is faced with the problem of SNR gain loss which is unfavorable for angle estimation. One of the solutions to alleviate the SNR gain loss is subarray MIMO radar. Subarray MIMO can be grouped into overlapped and non-overlapped cases according to the subarray configuration. In this paper, we focus on the type of the overlapped subarray configuration [9], which is different from the non-overlapped situation that antennas of transmit array are simply partitioned into subarrays without transmit coherent processing [10]. The overlapped transmit subarray (OTS) for MIMO radar has a transmit coherent gain which results in improvement in SNR per virtual element, while non-overlapped configuration does not have such a gain. In essence, this type of subarray MIMO radar can be regarded as a tradeoff between phased-array and MIMO radar, and more details could be found in [9]. The transmit array of the subarray MIMO radar is divided into several OTSs, and the transmit waveforms are coherent within each OTS while orthogonal to the waveform transmitted by other OTSs. In addition, transmit beam-pattern optimization with respect to the beamforming weights of the subarrays can be employed to focus transmitted energy and improve the SNR gain per virtual element [11]. However, transmit beampattern optimization is often a high computation work.

Angle estimation of multiple targets is one of the most important applications of radar system in practice. Some classic estimation algorithms, such as Capon, multiple signal classification (MUSIC) and Estimation of signal parameters via rotational invariance techniques (ESPRIT), have been applied to MIMO radar [5,10–17]. In bistatic MIMO radar, direction of arrival (DOA) and direction of departure (DOD) of multiple targets are obtained and paired automatically. Methods in [5,12] require an exhaustive peak search or root finding over the unknown parameters and hence bear high computational cost if the search is performed over a fine grid. The ESPRIT based methods proposed in [10,13–16] both take full advantage of the rotational invariance property of the uniform linear array and they are free of peak searches and achieves two-dimensional angle estimations. Moreover, [13] provides an effective ESPRIT scheme for multiple subarrays in monostatic MIMO, but its focus is on beam-pattern optimization, which is a high computation work. Methods in [10] and [16] are effective for just three transmit antennas configuration and only two non-overlapped subarrays with three or more transmit antennas, respectively. Unitary ESPRIT is applied in [17], which provides increased estimation accuracy with a reduced computational burden.

Among the methods above, the directional gain of transmit array of bistatic MIMO radar is not exploited. Without taking transmit directional gain into consideration, they all suffer from low SNR per virtual antenna as a result of dividing the total transmit energy over different waveforms without transmit coherent gain. In this paper, we propose a method of joint DOA and DOD of multiple targets present in the same range-bin for bistatic MIMO radar configured with multi-overlapped-transmit-subarray (MOTS), which we call MOTS MIMO radar for short. First, all the OTSs are partitioned into two groups which has a rotational invariance relationship. Then, a synthetic observation data matrix whose data samples can be doubled is built, in terms of the property of centro-Hermitian matrix. And the rotational invariance relationship obtained can be transformed into real-valued. Finally, the DOAs and DODs of targets can be solved in closed form in a new manner and paired automatically.

The rest of this paper is organized as follows. In Section 2, we briefly introduce the signal model of MOTS MIMO radar. In Section 3, a new unitary ESPRIT method is applied to MOTS MIMO radar. MOTS configuration systems are considered and the closed-form solution of angles is derived. In Section 4, the computational complexity of the proposed method is evaluated. The simulations results that show the advantages of the proposed scheme are presented in Section 5, which is followed by the conclusions in Section 6.

*Notation:* Scalars, column vectors and matrices are expressed by regular, bold lowercase and bold uppercase, respectively. (·)* (·)*^T^* (·)*^H^* and (·)^−1^ denote conjugate, transpose, conjugate transpose and matrix inverse, respectively. ***I****_N_* represents a *N* × *N* identity matrix, **Π***_MN_* stands for the *MN* × *MN* exchange matrix with ones on its antidiagonal and zeros elsewhere, *diag*(·) denotes a diagonalization operator and *E*[·] denotes the expectation operator. ⨂ and ⨀ represent Kronecker product and Hadamard product, respectively.

## 2. Signal Models of MOTS MIMO Radar

**Figure 1.**
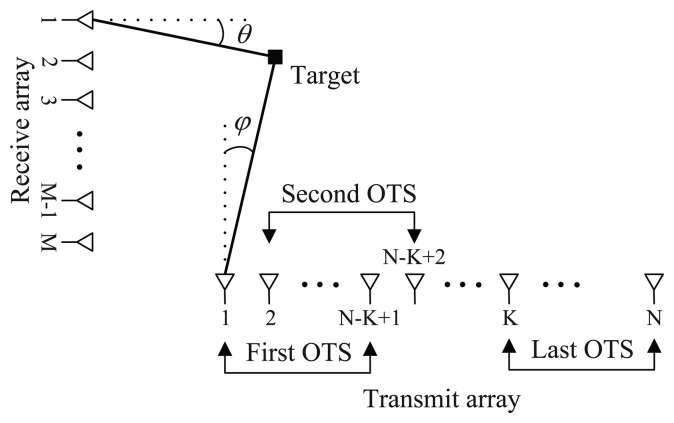
Multi-overlapped-transmit-subarray configured bistatic multiple-input multiple-output (MOTS MIMO) radar scenario.

Consider a bistatic MIMO radar system, shown in Figure 1, with *N* transmit antennas and *M* receive antennas, both of which are half-wave-length spaced uniform linear arrays (ULAs). The transmit array is partitioned into a total of *K* OTSs (2 ≤ *K* ≤ *N*), and the *k*th OTS contains *N_k_* = *N* − *K* + 1 array elements, *i.e.,* each OTS has the identical effective aperture. Let $\text{a}(\varphi )={\left[1,q_{r},q_{r}^{2},\ldots ,q_{r}^{N-1}\right]}^{T}$ with *q_r_* = e^−*jπ* sin(*φ*)^ and $\text{b}(\theta )={\left[1,q_{t},q_{t}^{2},\ldots ,q_{t}^{M-1}\right]}^{T}$ with *q_t_* = *e*^−*jπ* sin(*θ*)^ denote as transmit and receive steering vectors, respectively. ***a****_k_*(*φ*) denotes the steering vector associated with the *k*th OTS which is an *N_k_* × 1 subvector formed from the ***a***(*φ*). the waveform transmitted by the *k*th OTS is denoted by *ϕ_k_* (*t_l_*)*^T^* ∈ ℂ^1×^*^Q^* where *l* = 1, 2, …, *L*, *ϕ_k_* (*t_l_*)*^T^ϕ_k_* (*t_l_*) = 1, *ϕ_k_i__* (*t_l_*)*^T^ϕ_k_j__* (*t_l_*) = 0(*k_i_*, *k_j_* = 1, 2, …, *K*; *k_i_* ≠ *k_j_*), *t_l_* = *lT_r_* denotes the slow time where *T_r_* is the pulse repetition interval, *l* is the slow time index and *L* is the number of pulses. We assume that there are *P* targets at the same range bin with different Doppler frequencies. *f_p_* denotes Doppler frequency with *p* = 1, 2, …, *P*. ${\text{w}}_{k}=\frac{a_{k}(\varphi )}{\left\|a_{k}(\varphi )\right\|}$ donates the unit-norm complex vector of beamforming weights associated with the *k*th OTS. The signal reflected by the *p*th target located in the far-field can be then modeled as

(1) $$s_{T}^{r}(t_{l},\varphi_{p})=\sqrt{\frac{N}{K}}\sigma_{p}\sum_{k=1}^{K}{\text{w}}_{H}^{k}{\text{a}}_{k}(\varphi_{p})e^{-j\tau_{k}(\varphi_{p})}\phi_{k}{\left(t_{1}\right)}^{T}e^{j2\pi f_{p}t_{1}}$$

where *N* is the total transmitted energy for MOTS MIMO within one pulse, *σ_p_* is the reflection coefficient and *τ_k_* (*φ_p_*) is the time required for the wave to travel across the spatial displacement between the first element of the first OTS and the first element of the *k*th OTS.

Then, the *K* × 1 vector of the transmit coherent gain and waveform diversity vector can be introduced as follows,

(2) $$\text{c}(\varphi_{p})={\left[{\text{w}}_{1}^{H}{\text{a}}_{1}(\varphi_{p}),{\text{w}}_{2}^{H}{\text{a}}_{2}(\varphi_{p}),\ldots ,{\text{w}}_{K}^{H}{\text{a}}_{K}(\varphi_{p})\right]}^{T}$$

(3) $$\text{d}(\varphi_{p})={\left[e^{-j\tau_{1}(\varphi_{p})},e^{-j\tau_{2}(\varphi_{p})},\ldots ,e^{-j\tau_{K}(\varphi_{p})}\right]}^{T}$$

where ***c***(*φ_p_*) = [*N_k_*, *N_k_*, …, *N_k_*] ∈ ***R****^K^*^×1^, *N_k_* denotes the transmit coherent gain and $\text{d}(\varphi_{p})={\left[1,q_{t},q_{t}^{2},\ldots ,q_{t}^{K-1}\right]}^{T}$, which is an *K* × 1 subvector formed from the ***a***(*φ*). Then the reflected signal in Equation (1) can be rewritten as

(4) $${\text{S}}_{r}^{T}=\sqrt{\frac{N}{K}}\sigma_{p}{\left(\text{c}(\varphi_{p})⊙\text{d}(\varphi_{p})\right)}^{T}\left[\begin{array}{c} \phi_{1}{\left(t_{l}\right)}^{T}\\ \vdots \\ \phi_{K}{\left(t_{l}\right)}^{T} \end{array}\right]e^{j2\pi f_{p}t_{l}}$$

At the receive array, the array observations can be written as

(5) $$\text{X}=\sum_{p=1}^{P}\text{b}(\theta_{p}){\text{S}}_{r}^{T}$$

By match-filtering ***X*** to each of the waveforms ${\left\{\phi_{k}(t_{1})\right\}}_{k=1}^{K}$, we can form the output of the match filter within the duration of *L* pulses, which is corresponding to the *k*th OTS

(6) $${\text{Y}}_{k}=\sum_{p=1}^{P}\text{b}(\theta_{p})u_{k}(\varphi_{p})\sqrt{\frac{N}{K}}\sigma_{p}e^{j2\pi f_{p}t}={\text{BD}}_{k}\Phi +N_{k}$$

where ${\text{Y}}_{k}={\left[{\text{y}}_{1}^{T},\ldots ,{\text{y}}_{M}^{T}\right]}^{T}$, ***B*** = [***b***(*θ*_1_), …, ***b***(*θ_P_*)] ∈ ℂ^*M*×*P*^, ***D**_k_* = *diag* ([*u_k_*(*φ*_1_), …, *u_k_*(*φ_P_*)]*^T^*) ∈ ℂ^*P*×*P*^ with $u_{k}(\varphi_{p})=N_{k}q_{t}^{k-1}$ being the *k*th element of ***u***(*φ_p_*) = [***c***(*φ_p_*) ⨀ ***d***(*φ_p_*)]*^T^* ∈ ℂ^*K*×1^, $\Phi =\left[\begin{array}{c} \sqrt{\frac{N}{K}}\sigma_{1}e^{j2\pi f_{p}t}\\ \vdots \\ \sqrt{\frac{N}{K}}\sigma_{P}e^{j2\pi f_{p}t} \end{array}\right]\in {\mathbb{C}}^{P\times L}$, ***t*** = [*t*_1_, *t*_2_,…, *t_L_*] and ***N****_k_* is the received noise of the *k*th slice. It should be noted that the effective aperture of MOTS MIMO radar and traditional MIMO radar are (*M* + *K* − 2) *λ*/2 and (*M* + *N* − 2) *λ*/2 , respectively, where *λ* is the wave length. Accordingly, the SNR gain per virtual element of them are (*N_k_N*)/*K* and *N*/*M* [13]. For *K* < *N*, the MOTS MIMO radar has the smaller effective aperture as the traditional MIMO radar but higher SNR per virtual element due to the transmit coherent processing.

## 3. A Unitary ESPRIT Method for MOTS MIMO

In this section, the MOTS configuration, *i.e.*, the number of OTSs is *K*, is taken into account. From Equation (6) we can easily obtain data ***Y****_k_,* where *k* = 1, 2, …, *K*. After stacking the individual components ***Y****_k_* in one column, we obtain the following observation data matrix

(7) $$\text{Y}=\left[\begin{array}{c} {\text{y}}_{1}\\ {\text{y}}_{2}\\ \vdots \\ {\text{y}}_{K} \end{array}\right]=\left[\begin{array}{c} {\text{BD}}_{1}\Phi +{\text{N}}_{1}\\ {\text{BD}}_{2}\Phi +{\text{N}}_{2}\\ \vdots \\ {\text{BD}}_{K}\Phi +{\text{N}}_{K} \end{array}\right]=\left[\begin{array}{c} {\text{BD}}_{1}\\ {\text{BD}}_{2}\\ \vdots \\ {\text{BD}}_{K} \end{array}\right]\Phi +\left[\begin{array}{c} {\text{N}}_{1}\\ {\text{N}}_{2}\\ \vdots \\ {\text{N}}_{K} \end{array}\right]=\text{C}\Phi +N$$

where $C=\left[\begin{array}{c} C_{1}\\ C_{2}\\ \vdots \\ C_{K} \end{array}\right]$ denotes the steering matrix with ***C****_k_* = ***BD****_k_* and $N=\left[\begin{array}{c} N_{1}\\ N_{2}\\ \vdots \\ N_{K} \end{array}\right]$ denotes the observation noise matrix.

As seen from Figure 1, every *N_k_* elements of transmit array is used to form the identical OTS. All the OTSs are partitioned into two groups, which are composed of the first and last (*K* − 1) OTSs, respectively. From Equations (6) and (7), a rotational invariance relationship can be developed as

(8) $$J_{2}C=J_{1}C\Lambda_{t}$$

where ***J****_1_* and ***J***_2_ are *M*(*K* − 1) × *MK* block selection matrices, which are defined as ***J****_1_* = [***I**_M_*_(*K* − 1)_, **0**_*M*(*K* − 1)×*M*_] and ***J***_2_ = [**0**_*M*(*K* − 1)×*M*_, ***I***_*M*(*K* − 1)_], respectively. $\Lambda_{t}=D_{k+1}D_{k}^{-1}=\text{diag}({\left[e^{-j\pi \sin(\varphi_{1})},\ldots ,e^{-j\pi \sin(\varphi_{P})}\right]}^{T})$. ***J***_1_ and ***J***_2_ select the observation data corresponding to the first and second group of OTSs from ***Y***, respectively.

Then, we build a synthetic observation data matrix, whose data samples can be doubled. The synthetic data matrix defined as

(9) $$Z=[Y,{\text{Π}}_{MK}Y*{\text{Π}}_{L}]\in {\mathbb{C}}^{MK\times 2L}$$

It can be shown that ***Z*** is centro-Hermitian [18]. According to the theorem in [19], a centro-Hermitian matrix can be mapped to a real matrix. The real-valued matrix from the complex-valued ***Z*** via a unitary transformation can be obtained as:

(10) $$\Omega =Q_{MK}^{H}\left[Y,{\text{Π}}_{MK}Y*{\text{Π}}_{L}\right]Q_{2L}\in R^{MK\times 2L}$$

where the unitary transform matrix ***Q****_m_* of even and odd order are respectively defined as

(11) $$Q_{2m}=\frac{1}{\sqrt{2}}\left[\begin{array}{cc} I_{m} & jI_{m}\\ \Pi_{m} & -j\Pi_{m} \end{array}\right],Q_{2m+1}=\frac{1}{\sqrt{2}}\left[\begin{array}{ccc} I_{m} & 0_{m\times 1} & jI_{m}\\ 0_{m\times 1}^{T} & \sqrt{2} & 0_{m\times 1}^{T}\\ \Pi_{m} & 0_{m\times 1} & -j\Pi_{m} \end{array}\right]$$

Then the *P* dominant left singular vectors ***E****_s_* ∈ ***R***^*MK*×*P*^ of the real-valued matrix **Ω** can be computed through a real-valued singular value decomposition (SVD) of **Ω**. Alternatively, ***E****_s_* can be also computed through a real-valued eigenvalue decomposition (ED) of RT=ΩΩH=Re(QMKHYYHQMK/2L). There exists a nonsingular matrix ***T*** ∈ ***R***^*P*×*P*^ such that

(12) $$E_{S}=Q_{MK}^{H}\mathrm{CT}$$

where $Q_{MK}^{H}C$ is the real-valued steering matrix. Since ***Q****_MK_* is unitary, *i.e.*, $Q_{MK}Q_{MK}^{H}=I_{MK}$, it can be obtained from Equation (8) by premultiplying both sides by $Q_{M(K-1)}^{H}$, as follows:

(13) $$Q_{M(K-1)}^{H}J_{2}Q_{MK}Q_{MK}^{H}C=Q_{M(K-1)}^{H}J_{1}Q_{MK}Q_{MK}^{H}C\Lambda_{t}$$

For the *p*th target, the shift invariance relation in (13) can then be written as

(14) $$Q_{M(K-1)}^{H}J_{2}Q_{MK}Q_{MK}^{H}C=e^{-j\pi \sin(\varphi_{P})}Q_{M(K-1)}^{H}J_{1}Q_{MK}Q_{MK}^{H}C$$

Note that the two selection matrices satisfy **Π***_M_*_(_*_K_*_− 1)_ ***J***_1_**Π***_MK_* = ***J***_2_. As a result,

(15) $$\begin{array}{ll} Q_{M(K-1)}^{H}J_{1}Q_{MK} & =Q_{M(K-1)}^{H}{\text{Π}}_{M(K-1)}{\text{Π}}_{M(K-1)}J_{1}{\text{Π}}_{MK}{\text{Π}}_{MK}Q_{MK}\\ & =Q_{M(K-1)}^{T}J_{2}{\text{Π}}_{MK}Q_{MK}^{*}\\ & ={\left(Q_{M(K-1)}^{H}J_{2}Q_{MK}\right)}^{*} \end{array}$$

where the fact that ${\text{Π}}_{m}Q_{m}=Q_{m}^{*}$ and **Π***_m_***Π***_m_* with *m* being any integer is exploited.

Let ***K***_1_ and ***K***_2_ , respectively, be the real and imagin)ary parts of $Q_{M(K-1)}^{H}J_{2}Q_{MK}$ with K1=Re(QM(K−1)HJ2QMK) and K2=Im(QM(K−1)HJ2QMK). Then, with Equation (15) substituted into Equation (14), we can obtain:

(16) $$e^{j\pi \sin(\varphi_{P})/2}(K_{1}+jK_{2})Q_{MK}^{H}C=e^{-j\pi \sin(\varphi_{P})/2}(K_{1}-jK_{2})Q_{MK}^{H}C$$

According to the definition of the tangent function, Equation (16) can be rewritten as:

(17) $$K_{2}Q_{MK}^{H}C=\tan(-\pi \sin(\varphi_{P})/2)K_{1}Q_{MK}^{H}C$$

For all the *P* targets, the complex-valued rotational invariance relation in Equation (8) can be transformed into the real-valued manifold as follows:

(18) $$K_{2}Q_{MK}^{H}C=K_{1}Q_{MK}^{H}C\Psi_{t}$$

where **Ψ***_t_* = *diag* ([tan (−*π* sin (*φ*_1_)/2) , …, tan (−*π* sin(*φ_P_*)/2)]*^T^*) is a real-valued diagonal matrix. With Equation (12) substituted into Equation (18), it can be obtained as:

(19) $$K_{2}E_{S}=K_{1}E_{S}\gamma_{t}$$

where **γ***_t_* = ***T***^−1^**ψ***_t_****T***. **γ***_t_* can be computed as the least squares (LS) or the total least squares (TLS) solution of Equation (19). Eigenvalue decomposition of **γ***_t_* yields

(20) $${\hat{\psi }}_{t}=V^{-1}\gamma_{t}V$$

where **Ψ̂***_t_* = *diag* ([Ψ_1_, Ψ_2_, …, Ψ*_P_*] *^T^*) and ***V*** are composed of the eigenvalues and the corresponding eigenvectors of **γ***_t_*, respectively. The DODs of the targets are

(21) $${\hat{\varphi }}_{p}=-\arcsin(2\arctan(\psi_{p})/\pi )$$

Note that ***V*** is the estimate of ***T***^−1^. In terms of Equation (12), it can be obtained

(22) $$\hat{C}=Q_{MK}E_{S}V$$

through which the estimates of DOAs can be obtained. Here, we develop a closed-form solution of DOAs by an averaging method. Combining Equation (6) with Equation (7), we assume **ξ***_p,k_* = *β_p,k_****b*** (*θ_p_*) to be the *p*th column of ***C*^***_k_* ∈ ℂ^*M*×*P*^ which is the submatrix of ***C*^**. Then, $\hat{b}(\theta_{p})=\xi_{p,k}\beta_{p,k}^{*}/{\left|\beta_{p,k}\right|}^{2}$ has the same phases as the steering vector *u_k_*(*φ_p_*)***b***(*θ_p_*), where *u_k_*(*φ_p_*) is a complex constant corresponding to ***C****_k_* and varying with different *k*. Since *û_k_*(*φ_p_*)***b*^** (*θ_p_*) brings the estimation errors of two variables, we only consider the case of *k* = 1 when *u_l_* (*φ_p_*) is a real constant. Therefore, $\hat{b}(\theta_{p})=\xi_{p,1}\beta_{p,1}^{*}/{\left|\beta_{p,1}\right|}^{2}$ has the same phases as the steering vector ***b***(*θ_p_*). Let **Γ̂** = *angle* (*b̂*(*θ_p_*)) be the phases of ***b*^**(*θ_p_*). Thus, we can obtain the estimation of the DOAs via the method in [16] However, we can also obtain the estimation of the DOAs by virtue of the difference ***δ****_r_* between the adjacent elements of the **Γ̂**. The *i*th element of ***δ***_Γ_ can be obtained as

(23) $${\hat{\delta }}_{\Gamma }(i)=\hat{\Gamma }(i+1)-\hat{\Gamma }(i)$$

where *i* = 1, 2, …, *M* − 1. Under the ideal condition, the estimate of ***δ***_Γ_ can be given as

(24) $$\begin{array}{l} \delta_{\Gamma_{0}}={\left[0,\pi \sin(\theta_{p}),\ldots ,\pi \sin(\theta_{p})\right]}^{T}\\ =\Theta_{\delta }\sin(\theta_{p}) \end{array}$$

where **Θ***_δ_* = [0, *π*, …, *π*]*^T^* ∈ ***R***^(*M* − 1)×1^. Then the least-square estimation of the angle can be given as

(25) $$\begin{array}{ll} {\hat{\theta }}_{p} & =\arg\underset{\theta }{\min}{\left\|{\hat{\delta }}_{\Gamma }-\Theta_{\delta }\sin(\theta_{p})\right\|}^{2}\\ & =-\text{arcsin}\left(\frac{{\hat{\delta }}_{\Gamma }^{T}\Theta_{\delta }}{\Theta_{\delta }^{T}\Theta_{\delta }}\right)\\ & =-\text{arcsin}\left(\frac{1}{(M-1)\pi }\overset{M-1}{\underset{i=1}{\text{∑}}}{\hat{\delta }}_{\Gamma }(i)\right) \end{array}$$

It is shown that the DODs and DOAs of targets can be paired automatically.

## 4. Computational Complexity Analysis

In order to analyze the complexity for the computation of the proposed method, we have to know the complexity of the SVD algorithm, which is the main computational burden. Since the computational complexity of the SVD is varying with different methods [20]. Among them, an efficient orthogonal iteration approach for SVD algorithm has a complexity in order of *O*(*MNr*) for an *M* × *N* matrix truncated to rank *r*. Based on this point, the main computational cost of the ESPRIT method in [15] is *O* (*M*^2^*N*^2^*P*). Different from the *MN* × *MN* complex-valued covariance matrix in the ESPRIT method, the covariance matrix of the unitary ESPRIT in [17] with the same size *MN* × *MN* is real-valued.Since the complexity of complex-valued computation is higher than that of the real-valued, the computational complexity of the unitary ESPRIT in [17] is *O*(*μM*^2^*N*^2^*P*), where *μ* < 1. The computational complexity of our method is *O*(*μM*^2^*K*^2^*P*), which is approximately equal to unitary ESPRIT in [17] and lower than ESPRIT in [15].

## 5. Simulation Results

Consider a traditional bistatic MIMO radar which consists of a ULA of *M* = 8 receive antennas and a ULA of *N* = 6 omni-directional transmit antennas, which are spaced half a wavelength apart from each other. The *N* transmit antennas transmitting *N* orthogonal waveforms, and the *n*th waveform transmitted is the *n*th row of ***S*** ∈ ℂ^*Q*×*Q*^, where $S=(1+j)/\sqrt{2Q}H_{Q}$ and ***H****_Q_* is the *Q* × *Q* Hadamard matrix. Based on the configuration of the traditional bistatic MIMO radar, the MOTS MIMO radar improves its transmitter which has *K* OTSs transmitting *K* orthogonal waveforms and the *k*th transmitted waveform *ϕ_k_* (*t_l_*) is the *k*th row of ***S***. *Q* = 256 is the number of samples per pulse period, and *T_r_* = 64 *μs* is the pulse repetition interval. Assume that there exists *P* = 3 noncoherent sources, which are located at angles (*φ*_1_, *θ*_1_) = (20°,-20°) , (*φ*_2_, *θ*_2_) = (−18°, 10°) and (*φ*_3_, *θ*_3_) = ( 5°, 35°) , and the reflection coefficients of the targets are ${\left\{\sigma_{p}\right\}}_{p=1}^{3}=1$. The Doppler frequencies are ${\left\{f_{p}\right\}}_{p=1}^{3}=\{200,400,600\}\;Hz$. The additive noise is Gaussian zero-mean unit-variance spatially and temporally white. The average root mean square error (ARMSE) of the angle estimation is defined as:

(26) $$\text{ARMSE}=\sqrt{\frac{1}{P\eta }\overset{P}{\underset{p=1}{\text{∑}}}\overset{\eta_{\max}}{\underset{\eta =1}{\text{∑}}}\left[{\left({\hat{\varphi }}_{p}^{\eta }-\varphi_{p}\right)}^{2}+{\left({\hat{\theta }}_{p}^{\eta }-\theta_{p}\right)}^{2}\right]}$$

where *η*_max_ = 200 is the number of Monte Carlo trials and ${\hat{\varphi }}_{p}^{\eta }$ and ${\hat{\theta }}_{p}^{\eta }$ are the estimation of DOD *φ_p_* and DOA *θ_p_* of the *η*th Monte Carlo trial, respectively.

**Figure 2.**
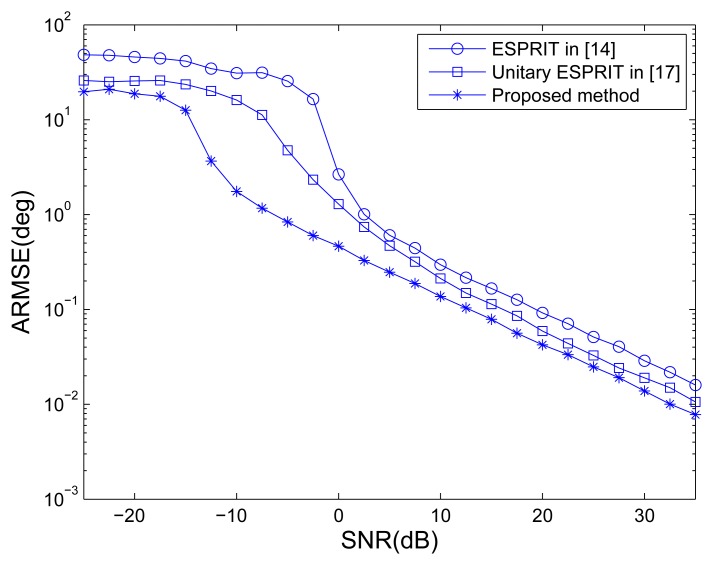
Average root mean square errors (ARMSEs) of angle estimation *versus* SNR.

**Figure 3.**
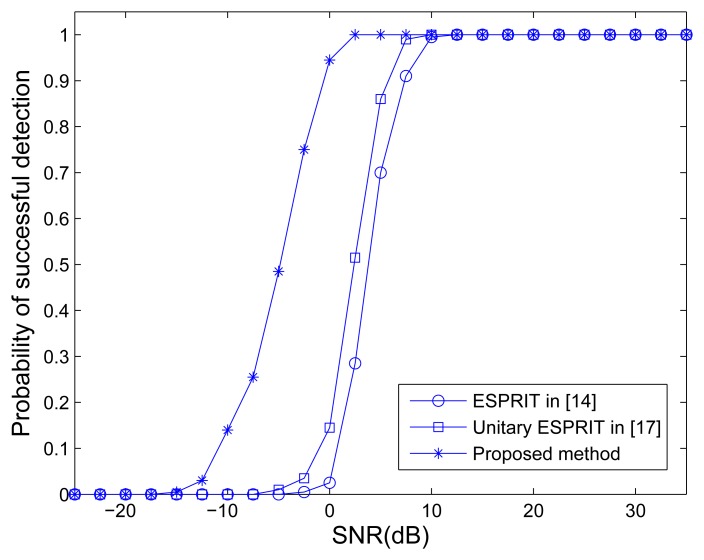
Probability of successful detection *versus* SNR.

The traditional MIMO radar employing ESPRIT [15] and unitary ESPRIT [17], and MOTS MIMO with *K* = 3 OTSs employing our method are used to compare. Figure 2 shows the ARMSE of angle estimation of different methods *versus* SNR, where *L* = 40. It can be seen that the traditional MIMO radar employing unitary ESPRIT has better performance of angle estimation than ESPRIT. It can also be observed that the traditional MIMO radar employing ESPRIT and unitary ESPRIT provide worse accuracy performance than the MOTS MIMO employing the proposed method. The MOTS MIMO radar has the smaller effective aperture as the traditional MIMO radar but higher SNR per virtual element due to transmit coherent processing. It yields the comparative advantage of the proposed scheme over traditional MIMO scheme, especially at lower SNR region. Figure 3 shows the probability of the successful detection of the different methods *versus* SNR. The absolute errors |*φ̂_p_* − *φ_p_*| ≤ 0.5**°** and |*θ̂_p_* − *θ_p_*| ≤ 0.5**°** are both required for the successful detection of DOD and DOA for all three targets. It can be seen from Figure 3 that all the methods exhibit a 100% successful detection at high SNR values. As the SNR decreases, the probability of successful detection starts dropping for each method at a certain point. The SNR level at which the transition happens is known as the SNR threshold. It also can be seen that MIMO employing ESPRIT and MIMO employing unitary ESPRIT have the highest and the second highest SNR thresholds, respectively. In contrast, the proposed method has the lowest SNR threshold, *i.e.*, the best probability of the successful detection.

**Figure 4.**
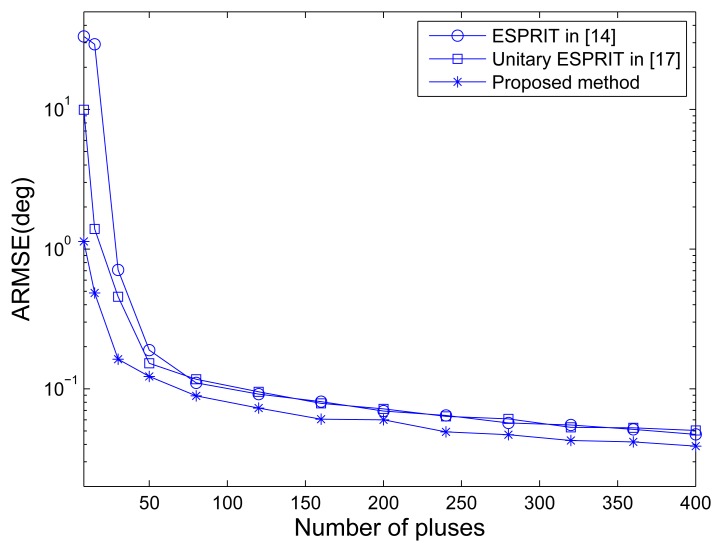
ARMSEs of angle estimation *versus* the number of pulses.

Figure 4 shows the RMSE of angle estimation of different methods *versus* the number of pulses under SNR = 10 dB. It is indicated that the unitary ESPRIT has better angle estimation performance than the ESPRIT in traditional MIMO configuration especially in small pulse number case. The performance improvement is lost for large pulse number, and this may be caused by the averaging of the number of pulses without SNR improvement. The MOTS MIMO employing the proposed method has the best performance of angle estimationin all pulse number cases, mainly due to the SNR improvement per virtual element via transmit coherent processing.

**Figure 5.**
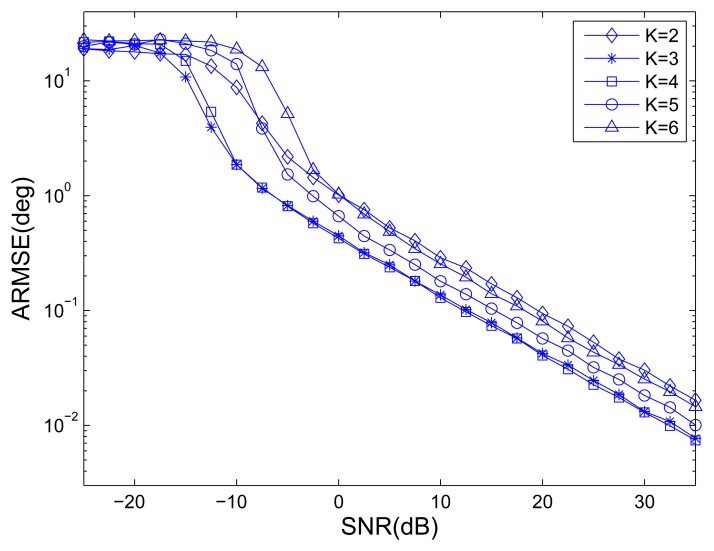
ARMSEs of angle estimation *versus* the number of overlapped-transmit-subarrays (OTSs).

For different numbers of OTSs, Figure 5 shows the ARMSE of angle estimation of MOTS MIMO employing the proposed method *versus* SNR , where *L* = 40. Note that if *K* = 1 is chosen, the whole transmit array is considered as one subarray and only one waveform is emitted, there is no estimation of DODs. When *K* = 6 is chosen, the MOTS MIMO becomes the MIMO radar without subarray partitioning, which has largest effective aperture but no transmit coherent gain. It can be easily seen that the angle estimation performances are the best when the number of OTSs is equal to 3 or 4. It is the fact that effective aperture becomes larger with an increase in the number of OTSs *K* while SNR gain per virtual antenna becomes lower. It is the combination of effective aperture and SNR gain per virtual antenna that has an impact on the estimation performance. It also can be observed that the MOTS MIMO with *K* = 3 slightly outperforms the MOTS MIMO with *K* = 4 at low SNR region while the opposite occurs at high SNR region. This means that having high SNR gain per virtual antenna is more important at low SNR region, while having large effective aperture is more important at high SNR region. Thus, *K* is chosen as the tradeoff between effective aperture and SNR gain per virtual antenna.

## 6. Conclusions

In this paper, a unitary ESPRIT scheme is presented to DOD and DOA estimation for MOTS MIMO radar. The proposed method exploits the combination of inherent advantages of MOTS MIMO radar with unitary ESPRIT. The MOTS MIMO improves the SNR gain through the carefully chosen number of OTSs, which is a tradeoff between effective aperture and SNR gain per virtual antenna. The proposed method, which is based on unitary ESPRIT, doubles the data samples and reduces the computational burden. Our scheme for MOTS MIMO provides increased estimation accuracy of angle estimation with DODs and DOAs of targets solved in closed form and automatically paired in a simple manner. Several experimental results have demonstrated the performance of the proposed scheme.

## References

[1] Fishler E., Haimovich A., Blum R., Chizhik D., Cimini L., Valenzuela R. MIMO radar: An idea whose time has come.

[2] Li J., Stoica P. MIMO radarâᾸŞdiversity means superiority.

[3] Haimovich A.M., Blum R.S., Cimini L.J. (2008). MIMO radar with widely separated antennas. IEEE Signal Process. Mag..

[4] Bekkerman I., Tabrikian J. (2006). Target detection and localization using MIMO radars and sonars. IEEE Trans. Signal Process..

[5] Yan H., Li J., Liao G. (2008). Multitarget identification and localization using bistatic MIMO radar systems. EURASIP J. Adv. Signal Process..

[6] Bliss D.W., Forsythe K.W. Multiple-input multiple-output (MIMO) radar and imaging: Degrees of freedom and resolution.

[7] Li J., Stoica P., Xu L., Roberts W. (2007). On parameter identifiability of MIMO radar. IEEE Signal Process. Lett..

[8] Robey F.C., Coutts S., Weikle D., McHarg J.C., Cuomo K. MIMO radar theory and experimental results.

[9] assanien A., Vorobyov S.A. (2010). Phased-MIMO radar: A tradeoff between phased-array and MIMO radars. IEEE Trans. Signal Process..

[10] Chen J., Gu H., Su W. (2010). A new method for joint DOD and DOA estimation in bistatic MIMO radar. Signal Process..

[11] Ren S., Ma X., Yan S., Hao C. (2013). 2-D unitary ESPRIT-like direction-of-arrival (DOA) estimation for coherent signals with a uniform rectangular array. Sensors.

[12] Zhang X., Xu L., Xu L., Xu D. (2010). Direction of departure(DOD) and direction of arrival (DOA) estimation in MIMO radar with reduced-dimension MUSIC. IEEE Commun. Lett..

[13] Hassanien A., Vorobyov S.A. (2011). Transmit energy focusing for DOA estimation in MIMO radar with colocated antennas. Signal Process..

[14] Duofang C., Baixiao C., Guodong Q. (2008). Angle estimation using ESPRIT in MIMO radar. Electron. Lett..

[15] Jinli C., Hong G., Weimin S. (2008). Angle estimation using ESPRIT without pairing in MIMO radar. Electron. Lett..

[16] Jin M., Liao G., Li J. (2009). Joint DOD and DOA estimation for bistatic MIMO radar. Signal Process..

[17] Zheng G., Chen B., Yang M. (2012). Unitary ESPRIT algorithm for bistatic MIMO radar. Electron. Lett..

[18] Haardt M., Nossek J.A. (1995). Unitary ESPRIT: How to obtain increased estimation accuracy with a reduced computational burden. IEEE Trans. Signal Process..

[19] Lee A. (1980). Centrohermitian and skew-centrohermitian matrices. Linear Algebra Appl..

[20] Golub G.H., Van Loan C.F. (1996). Matrix Computations.

